# TRAIL receptor-induced features of epithelial-to-mesenchymal transition increase tumour phenotypic heterogeneity: potential cell survival mechanisms

**DOI:** 10.1038/s41416-020-01177-w

**Published:** 2020-12-01

**Authors:** Ludovic Peyre, Mickael Meyer, Paul Hofman, Jérémie Roux

**Affiliations:** Université Côte d’Azur, CNRS UMR 7284, Inserm U 1081, Institut de Recherche sur le Cancer et le Vieillissement de Nice (IRCAN), Centre Antoine Lacassagne, 06107 Nice, France

**Keywords:** Metastasis, Tumour heterogeneity

## Abstract

The continuing efforts to exploit the death receptor agonists, such as the tumour necrosis factor (TNF)-related apoptosis-inducing ligand (TRAIL), for cancer therapy, have largely been impaired by the anti-apoptotic and pro-survival signalling pathways leading to drug resistance. Cell migration, invasion, differentiation, immune evasion and anoikis resistance are plastic processes sharing features of the epithelial-to-mesenchymal transition (EMT) that have been shown to give cancer cells the ability to escape cell death upon cytotoxic treatments. EMT has recently been suggested to drive a heterogeneous cellular environment that appears favourable for tumour progression. Recent studies have highlighted a link between EMT and cell sensitivity to TRAIL, whereas others have highlighted their effects on the induction of EMT. This review aims to explore the molecular mechanisms by which death signals can elicit an increase in response heterogeneity in the metastasis context, and to evaluate the impact of these processes on cell responses to cancer therapeutics.

## The epithelial-to-mesenchymal transition (EMT) features

EMT is a physiological process that occurs during embryogenesis (type 1 EMT), wound healing phases (type 2 EMT) and metastasis (type 3 EMT). In these distinct situations where it undertakes development,^1^ cellular homoeostasis and repair,^2^ EMT is characterised by the loss of some epithelial features and, in parallel, by the gain of new mesenchymal properties such as the acquisition of invasive capacities and resistance to apoptosis. The newly acquired phenotype has stem cell capacities that confer pluripotency and plasticity to cells, but also a different sensitivity to both endogenous and environmental signals.^3^ It is a critical process for tumour initiation and progression.^4^

During EMT, E-cadherin, the main cadherin responsible for epithelial cell adherent junctions, but also a regulator of actin cytoskeleton homoeostasis and organisation,^5,6^ is downregulated primarily via transforming growth factor (TGF)-β/SMAD signalling, leading to a loss of cell–cell adhesion.^7^ To date, cancer cells with a low level of E-cadherin are considered invasive (or aggressive), whereas those with a high level are associated with stress resistance and survival.^8^ However, E-cadherin expression has recently been shown to be crucial for metastasis by preventing reactive oxygen species (ROS)-dependent cell death and by allowing cancer cell dissemination,^9^ a finding in accordance with clinical evidence showing metastatic E-cadherin^+^ tumours.^10,11^

Pathways other than TGF-β/SMAD also play a central role in relaying the EMT signal. This is the case for receptor tyrosine kinases (RTKs), Notch, Hedgehog and the canonical and the non-canonical Wnt pathways.^12^ They all activate EMT-inducing transcription factors (EMT-TFs). Among them, the Snail family (Snail and Slug), ZEB-1/2, TWIST1/2, TCF3, FOXC2, PRRX1, YAP/TAZ and SOX4/9 target E-cadherin repression or co-operate with core EMT-TFs.^13^ Because EMT-TFs are differentially expressed depending on the cancer stage, as observed, for example, in endometrioid endometrial carcinoma,^14^ the spatiotemporal expression of the EMT-TF TWIST1 can be a mechanism for the hierarchical role of EMT-TFs observed during cancer progression,^15^ whereas the miR-34/SNAIL and the miR-200/ZEB axis not only regulates E-cadherin expression, but also the hybrid phenotype via a double-negative feedback.^16–19^

Evidence concerning the acquisition of stem cell characteristics associated with the induction of partial EMT, also known as a hybrid E/M state, has been described and is associated with an increase in tumour-propagating cell (TPC) frequency.^20^ By showing that the earliest EMT state can exhibit a high TPC frequency, the authors demonstrated that intermediate states can also provide stem cell properties, leading to drug resistance and cancer progression, and this mechanism does not require the establishment of a full EMT as it had been assumed previously. Indeed, a report that EMT was not required for lung metastasis was based on the observation that metastasis in secondary sites mostly exhibited an epithelial phenotype.^21^ However, the actual tools and methods used to claim such a controversial conclusion have been questioned and found to be insufficient to rule out the EMT process during cancer progression.^22^ Therefore, the hybrid E/M system is still the proposed mechanism by which EMT drives metastatic dissemination.^23–25^

Cell death resistance has been shown in partial EMT states,^26^ and the hybrid E/M phenotype has been described in tumours as a source of cancer cell response heterogeneity with differences in sensitivity to apoptotic stimulus such as anoikis and anticancer drugs.^27,28^ The main signalling pathways involved in the development and regenerating processes are also involved in this mechanism in neoplastic tissue. Among them, the Notch/Jagged pathway stabilises the hybrid E/M phenotype and is necessary to expand the fraction of cancer stem cells (CSCs). This has been shown in a triple-negative breast cancer model under the influence of interleukin (IL)-6, a pro-inflammatory cytokine able to activate the EMT programme.^29^ Still in the mammary gland, the EMT programme also increases stem-like features through the control of the Hedgehog signalling pathway,^30^ whereas Wnt pathways regulate the stem cell programme of hybrid E/M phenotypes that accounts for increasing drug resistance.^31–33^

CSCs have tumorigenic potential that depends on the EMT state.^33^ EMT also confers stem cell properties to cancer cells by inducing non-genetic and heritable epigenetic changes;^34^ however, these newly acquired properties are known to be reversible through the induction of a mesenchymal-to-epithelial programme called MET,^35^ with the EMT/MET switch contributing to cell phenotypic plasticity.^36,37^ Because a large number of phenotypic states exist between partial and the well-differentiated states of EMT, this phenotypic diversity thus increases intra-tumoural heterogeneity^38^ and is a potential source of drug resistance observed in patients.^39^

### EMT and cell survival

Evidence that EMT not only provides the mechanistic basis of metastasis, but also of resistance to apoptosis, has been demonstrated in different model tissues, such as breast,^40^ lung,^21^ prostate^41^ or pancreas,^42^ with a process potentially dependent on miR-200,^43,44^ TWIST and Snail1 expression.^45,46^ The decreased expression of key proteins, such as cadherins and integrins, is accompanied by the loss of cell adhesion with the extracellular matrix (ECM) and with neighbouring cells during the EMT process. These lead to the activation of intracellular pro-survival signals known as ‘non-canonical pathways’ and is mainly mediated by the phosphoinositide 3-kinase (PI3K)/Akt (also known as protein kinase B (PKB)) pathway.

The PI3K/Akt signalling pathway plays a pivotal role in controlling cancer cell survival. More specifically, it allows the activation of the mitogen-activated protein kinase (MAPK) pathway responsible for the activation of downstream p90RSK, thus inhibiting the pro-apoptotic protein Bad.^47^ In prostate cancer, PI3K/Akt signalling is activated downstream by the involvement of Notch and activation of the Hedgehog pathway. While Hedgehog increases the expression of the anti-apoptotic Bcl-2, Notch mediates pro-survival mechanisms under the control of Akt, thus leading to docetaxol resistance.^48^ Akt activation also drives nuclear factor (NF)-κB activation, which, in turn, controls the expression of the anti-apoptotic proteins FLICE-inhibitory protein (FLIP) and X-linked inhibitor of apoptosis protein (XIAP).^49^

The main EMT-TFs responsible for migration, invasion or dedifferentiation also play a role in cell survival by modulating the expression of pro- and anti-apoptotic proteins. For example, Twist increases Bcl-2, leading to apoptotic resistance,^50^ whereas SNAI1 interacts with poly(ADP-ribose) polymerase 1 (PARP1).^51,52^ Other apoptotic regulators are involved in EMT-dependent survival mechanisms. Among them, TGF-β, a tumour repressor with a dual role in cancer that depends on the environmental conditions such as matrix rigidity^53^ or cancer progression stage,^54^ has been shown to induce apoptosis and to interplay with the PI3K/Akt pathway.^55^

In conclusion, many environmental factors derived from the ECM, cancer-associated fibroblasts (CAFs), immune cells and vessels are responsible for the increase in EMT-TFs described above and are involved in multidrug resistance (MDR) phenomena. They not only regulate the expression of pro- or anti-apoptotic proteins, but also those of ABC transporter genes.^56^ Moreover, signals that induce EMT such as TGF-β could modulate the response of cancer cells to anticancer drugs (as with endogenous antimitotic signals) by cytokinesis failure, a heritable mechanism that leads to genomic instability.^57^ The dual role of TGF-β is applied in this context and can lead to opposite effects, depending on the cancer mutations and the model studied. For example, in Ras-mutant pancreatic cancer cells, sensitivity to apoptosis is then controlled by the TGF-β-induced EMT. In other words, TGF-β induces EMT and subsequent apoptosis confers a tumour-suppressive property to the EMT programme.^58^

## Death receptor-mediated features of EMT

Activation of transmembrane receptors of the tumour necrosis factor superfamily (TNF-SF), such as Fas/CD95, TNF receptors 1 and 2 (TNF-R1/R2) and TNF-related apoptosis-inducing ligand (TRAIL) receptors 1 and 2 (TRAIL-R1/2, DR4/DR5), by their respective ligands Fas ligand (Fas-L), TNF-α and APO-2L/TRAIL (Table 1), can lead to the induction of cell death.^59,60^ The binding of the ligand to its receptor allows the formation of a death-inducing signalling complex (DISC), including caspase-8,^61^ which can transduce a pro-apoptotic signal via the caspase cascade, leading to cell death.^62^

**Table 1:** EMT-associated molecular features of TRAIL receptors.

Death receptor	Alternative names	Functions	Pathways involved	EMT-associated molecular features	References
DR4	TRAIL-R1, Apo2, *TNFRSF10A*	Pro-apoptotic, Pro-survival	TAK1, MAPKs, NF-κB and caspases-8/10	Claudin and occludin (associated with FADD at the DISC), E-cadherin and FAT1 (protein interactions)	^72–74,79,81,82,97^
DR5	TRAIL-R2, *TNFRSF10B*				
DcR1	TRAIL-R3, *TNFRSF10C*	Anti-apoptotic	–	–	–
DcR2	TRAIL-R4, *TNFRSF10D*	Anti-apoptotic	_	Associated with N-cadherin overexpression	^89^
DcR3	TR6, M68, *TNFRSF6B*	Anti-apoptotic	–	–	–
OPG	OCIF, PDB5, *TNFRSF11B*	Anti-apoptotic	–	–	–

Claudin, occludin and E-cadherin are described as DR4/5-positive regulators, whereas FAT1 is considered a negative regulator. N-cadherin is a positive DcR2 regulator.

**Fig. 1:**
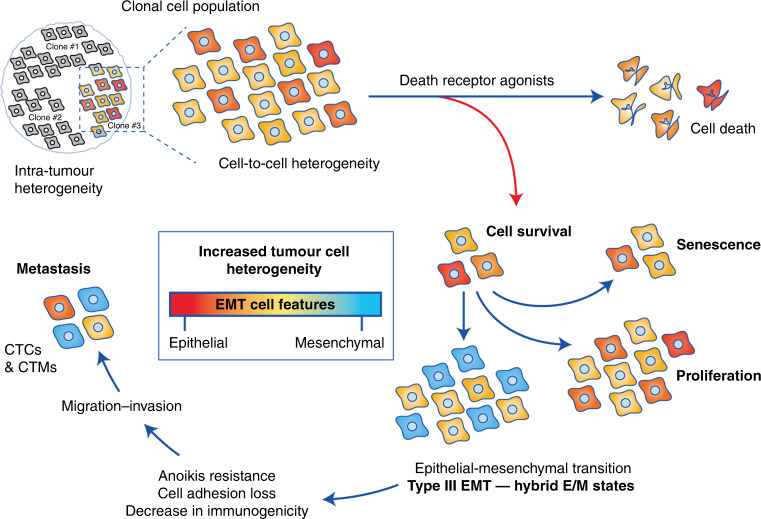
Effects of death receptor agonists on EMT-mediated cancer-cell heterogeneity. Binding of death ligands can activate pathways, including caspase-8-dependent apoptosis and survival. Cancer cells that survive treatment can give rise to different responses, such as proliferation, senescence or differentiation. Epithelial-to-mesenchymal transition (EMT) is one of the cell biological processes that contributes to cellular plasticity, allowing cancer cells to switch from an epithelial state to a mesenchymal one. Cells lose their adhesion capacities, acquire stem cell characteristics (CSCs) and can migrate until invading secondary sites via the lymphatic system and the blood circulation. EMT also provides resistance to anoikis, an apoptotic process following loss of cell contacts with ECM and decreases the immunogenic response. Together, these events can participate in the increased resistance of circulating tumour cells (CTCs) alone or in clusters (CTMs), allowing cancer progression and metastasis. Finally, EMT increases response heterogeneity by enhancing cell diversity within the tumour, which can further increase clonal heterogeneity and cancer cell resistance to chemotherapies.

In addition to apoptosis, a range of cell responses are induced upon binding to death receptors. Among them, differentiation was shown to be regulated by TRAIL-induced caspase activation in intestinal cells,^63^ osteoclasts^64^ and keratinocytes.^65^ A close relationship exists between differentiation steps during the early stage of development and cancer progression, leading to metastasis, and involves common molecular factors and pathways,^66^ such as death receptor activation or dysregulation. Indeed, metastasis and invasion are processes associated with TRAIL treatment and are shown to be dependent on the NF-κB pathway.^67^ In human cholangiocarcinoma cell lines, TRAIL promotes cell migration and invasion under the control of the NF-κB-dependent pathway.^68^ As cancer progression can be initiated through the induction of EMT, involving different cell differentiation steps, it is important to better understand the molecular mechanisms leading to the acquisition of heterogenous EMT features upon death receptor engagement, which have a further impact on the cell response (Fig. 1).

### Death receptors co-operate with cell membrane components: TRAIL and loss of cell adhesion

TRAIL has been reported to induce the loss of adhesion followed by drug resistance in several cellular contexts. First, through the apoptotic pathways, TRAIL activates the cleavage of substrates involved in cell differentiation and remodelling.^69^ Then, by interacting with cadherins at the plasma membrane (Table 1), DR4/5 stabilisation and activation can be impaired, leading to changes in TRAIL sensitivity, as discussed below.

Occludin and claudin, two transmembrane proteins forming tight junctions (TJs), play a barrier role not only in controlling fluid transport but also in proliferation and differentiation of the epithelium. As an epithelial marker, occludin is downregulated during EMT.^70^ Epigenetic silencing of occludin leads to metastasis of cancer cells via modulation of unique sets of apoptosis-associated genes.^71^ Moreover, its knockdown decreases TRAIL-induced cell death, thus conferring an important role of occludin in apoptosis.^72^ Indeed, occludin (and to a lesser extent claudin) interacts physically with Fas associated via death domain (FADD) and DISC when tight junctions are disrupted, a phenomenon allowing cell defence (an antibacterial strategy) by activating the extrinsic cell death signal.^73,74^

Studies have reported that distribution of TRAIL receptors in lipid rafts can be linked to cell sensitivity to TRAIL ligand,^75–78^ but how they interact with membrane components remains poorly understood. First, DR4/5 can couple with E-cadherin.^79^ Secondly, E-cadherin/α-catenin linkage with dynamic cytoskeleton is essential for the efficient assembly of active death receptor complexes.^80^ Consequently, this receptor clustering allows formation of active TRAIL signalling complexes and sensitises some cancer cells to death induced by TRAIL. Because EMT leads to the dysregulation and disassembly of this E-cadherin–TRAIL complex, cancer cells with a mesenchymal phenotype increase their protection against TRAIL-induced apoptosis.^81^ However, in patients with early-detected colorectal cancer, DR4 and DR5 can be expressed in parallel with E-cadherin, but their co-localisation at the membrane is not systematic.^82^ Moreover, the potential interactions between the death receptors (including the decoy receptors known as death receptor competitors that lack the intracellular death domain responsible for the propagation of TRAIL-induced apoptotic signal) and cadherins remains unanswered, despite the potential mechanistic impact these cell processes hold.^83^

A study focusing on the natural anti-metastatic agent antrocin has shown that it could act as an EMT inhibitor, restoring E-cadherin protein levels in parallel with the increase in DR5 expression,^84^ whereas another study has shown that DR5 knockdown could increase E-cadherin expression and diminish migration in breast cancer, which further suggests a specific regulatory step.^85^ However, how DR4/DR5 and E-cadherin expression is simultaneously regulated is still not well understood. Nevertheless, the Hedgehog pathway and the modulation of some microRNAs (miRNAs) may be involved in this regulatory process. Indeed, TRAIL-induced apoptosis resistance in chronic conditions has been shown to be under the control of miR-21, miR-30c and miR-100 in lung cancer.^86^ The overexpression of these miRNAs inhibits the expression of caspases-3/8 and the EMT marker E-cadherin. They also activate the NF-κB pathway that regulates via a feedback loop the expression of the miRNAs involved. Among them, miR-21 seems to play a pivotal role in modulating the expression of both E-cadherin and DR4/DR5 as observed in a glioblastoma model.^87^ In this study, the Hedgehog inhibitor NPV-LDE-225 blocked the EMT process and allowed an increase in the efficiency of TRAIL-induced apoptosis by increasing DR4/DR5 and E-cadherin expression. Finally, it decreases not only miR-21 levels, but also stem-cell markers such as NANOG, OCT4, SOX2 and c-Myc, making both miR-21 and the Hedgehog signalling pathway possible master regulators of this mechanism. In addition, the natural Hedgehog inhibitor cordycepin can also induce apoptosis in breast cancer models, with the upregulation of DR4/DR5 and E-cadherin.^88^

More recently, the co-operation of cadherin/DR was studied in head and neck cancers (HNSCCs): N-cadherin, the major mesenchymal marker, has been shown to enhance cell growth by inhibiting apoptosis.^89^ N-cadherin overexpression was associated not only with an increase in DcR2 but also with a decrease in DR5, whereas its knockdown led to the opposite results, suggesting the existence of a signalling network between cadherins and death receptors. Moreover, N-cadherin was observed to interact with DcR2 in these same models, a process allowing cell survival via cleavage of caspases by activating the MAPK/extracellular signal-regulated kinase (ERK) pathway. Because the E-/N-cadherin switch is the hallmark of EMT^90,91^ and because cell sensitivity to TRAIL is changing with cell status (from epithelial to mesenchymal, and this could represent, in turn, a TRAIL-sensitivity marker. Further investigations are needed to clarify this possible regulation even if limited data exist in the literature supporting this regulatory process. Indeed, we know that both DR5 and DcR2 expression are under the control of P53, and a negative-feedback loop has been proposed between DcR2 and P53.^92^ Overexpression of P53 can lead to an increase in DcR2 which, in turn, can attenuate DR5-mediated apoptosis.^93^

While E-cadherin co-operates with DR5 at the membrane level of epithelial cancer cells and N-cadherin with DcR2 in mesenchymal cells, another member of the cadherin family has been described to physically interact with TRAIL receptors, namely FAT1. FAT1 is a cadherin-like protein with tumour-suppressor functions, which plays a central role in developmental processes and cell communication.^94,95^ This adhesive molecule is highly expressed in several foetal epithelia, but its mutation leads to an aberrant activation of the WNT signalling pathway resulting in tumorigenesis.^96^ In glioblastoma cells, FAT1 acts as a negative regulator of DR4/5. After knockdown of FAT1, cancer cells became more sensitive to TRAIL-induced apoptosis, a process very similar to those mentioned above: by interacting with FAT1, DRs finally prevent DISC activation.^97^

Death receptors co-operate physically with other membrane proteins not specifically involved in EMT, but also involved in cancer progression, leading to metastasis. For example, carcinoembryonic antigen (CEA, CD66e), mainly found in colorectal cancer, is a cell-surface glycoprotein that is increased along with DR5 when cells are in suspension. Interestingly, it binds and inhibits DR5, resulting in the decreased activity of caspase-8. An increase in cell survival (in vitro) and colonisation of secondary tissues (in vivo) were also observed. Together, these events stimulate cancer progression and metastasis.^98^

### EMT regulates TRAIL sensitivity

Targeting mesenchymal cancer cells by displaying stem cell characteristics with TRAIL has been proposed to reduce resistance in different cancers, such as squamous and adenocarcinoma lung cancer.^99^ This association is emerging in other pathologies such as biliary atresia, a common viral-dependent cholangiopathy where EMT has been shown to block biliary innate immune response via TRAIL-mediated apoptosis^100,101^ or in hepatitis B virus (HBV) infection, where HBV may activate in certain conditions an EMT-like state that is ER-stress dependent.^102^

Interestingly, some homologies have been observed between differential sensitivity to TRAIL and the EMT process. TRAIL resistance of non-genetic origins from variable activation and expression levels of pro- and anti-apoptotic proteins^103**–**105^ has been shown to be transient and sustainable.^106^ Similar observations have been made in the EMT context. Indeed, during cell division, variable partitioning of macromolecules in daughter cells was proposed to increase EMT heterogeneity,^107^ illustrating that non-genetic mechanisms play an important role in cellular heterogeneity and plasticity, leading to different cell states. Because cancer cells can switch from an epithelial state to a mesenchymal one in order to adapt to the tumour microenvironment and to progress to metastasis, the intermediate states known as hybrid E/M linked to differences in sensitivity to chemotherapeutic agents^108^ are now emerging as promising targets against cancer progression.^109^

One of the first observations was related to nitric oxide (NO) donors such as DETANONOate. This chemical can sensitise cancer cells to TRAIL-induced apoptosis through different mechanisms. First, it contributes to increase the expression of Raf kinase inhibitor protein (RKIP), a metastatic tumour suppressor. Then, it inhibits both the NF-κB pathway responsible for cell resistance to chemotherapies and the YY1 transcription factor, which is, in turn, responsible for the regulation of Fas and DR5 (the main receptor for TRAIL). Finally, NO donors contribute to the inhibition of the Snail transcription factor, an E-cadherin repressor, thus repressing the EMT process. In brief, by dysregulating the NF-κB/Snail/YY1/RKIP/phosphatase and tensin homologue (PTEN) axis, NO donors prevent metastatic potential and resistance to apoptosis.^110,111^ Similar observations have been found in urothelial cancer cell lines where mesenchymal cells showed higher resistance to TRAIL treatments than epithelial cells. Indeed, the latter have a lower level of XIAP and Bcl-2 proteins that account in part for the anti-apoptotic effects. These data appear to be an additional point in favour of the importance of targeting EMT markers and/or processes as a strategy against cancer progression.^112^

A compelling observation suggesting a link between EMT and resistance to TRAIL-induced apoptosis is the deregulation of transcription factors such as Snail and Slug.^45,113^ Both are not only involved in the downregulation of adherent proteins known as epithelial markers such as E-cadherin, claudins or occludins, but also in the inhibition of pro-apoptotic proteins such as Bcl-2, Bid, Puma and caspase-9. Moreover, the upregulation of Snail and Slug leads to the increase in P53 protein levels that mediate resistance through anoikis.^114^ Thus, reverting EMT appears to be a strategy to sensitise cancer cells to TRAIL therapy. Srivastava et al. used a benzamide histone deacetylase inhibitor (MS-275 also called entinostat) to target histone deacetylase (HDAC) 1/3, leading to an increase in the apoptosis-inducing potential of TRAIL in different cancer cell lines in vitro.^113^ This treatment enabled to sensitise TRAIL-resistant cancer cells, a phenomenon also observed in vivo (breast cancer xenografts in nude mice) where MS-275 inhibits EMT, decreases NF-κB pathway activation and finally increased DR4/DR5 receptor and pro-apoptotic protein expression. In pancreatic CSCs, the same team demonstrated that a GLI transcription factor inhibitor (GANT-61), which targets the Hedgehog pathway, allowed EMT inhibition in parallel with an increase in DR4 and DR5 expression.^115^

Another mechanism proposed previously is the dysregulation of miRNAs, especially Mir-9, which has been found to be downregulated in many cancers.^116^ This miRNA can modulate the expression of interferon (IFN)-induced genes and MHC class I molecules. Among these IFN-induced genes, TRAIL has been shown to be one of them. Indeed, an increase in Mir-9 is associated with overexpression of TRAIL.^117^ TRAIL overexpression was also found in MCF-7 cancer cells that have acquired resistance to metformin treatment. By inducing autophagy in certain cancer cells, TRAIL can protect cells by blunting the cytotoxicity of the treatment, thus contributing to TRAIL resistance.^118^ Mir-9 is also known to interact with the TGF-β signalling pathway during EMT;^119^ however, information is still lacking about TRAIL sensitivity. It has only been reported that TGFβ-induced EMT plays a critical role during irradiation of the breast cancer cell line HMLE, leading to radioresistance of the stem-like breast cancer cells generated. Indeed, in this study, mesenchymal CD24^−/low^/CD44^+^ CSCs were shown to exert apoptosis resistance through differential activation of death receptors such as TRAIL and in parallel via the increased expression of the anti-apoptotic marker survivin.^120^ The changes observed in TRAIL gene expression are likely to be associated with an EMT signature in such cases. Furthermore, another miRNA candidate has been proposed to play such an important role in TRAIL-induced apoptosis resistance. For example, by downregulating the PI3K/Akt regulator PTEN, miR221 induces EMT and invasiveness of breast cancer cells.^121^

Lu et al. proposed a mechanism of EMT-dependent inhibition of apoptosis where loss of E-cadherin (which binds selectively to DR4 and DR5 but not to Fas owing to the DISC formation and caspase-8 activation) drives cancer-cell resistance to TRAIL treatment.^79^ Another study reported that EMT reversal by ML327, an isoxazole-based small-molecule probe that represses E-cadherin levels and partially reverses the EMT phenotype, is accompanied by an enhanced response to TRAIL in carcinoma cells and this was in an E-cadherin-independent manner.^122^

Involvement of the mitochondrial pathway in models such as melanoma is also critical in TRAIL sensitivity,^123^ but its relationship with EMT remains less well described. In lung cancer, when the EMT marker MUC1 (responsible for pro-oncogenic signal transduction) is silenced, TRAIL treatment becomes more efficient. This increased sensitivity is possibly due to the MUC1–BAX association, leading to prevention of mitochondrial permeabilisation in response to apoptotic stimuli.^124^

Depending on the EMT status and on the expression levels of pro- and anti-apoptotic proteins under the control of the EMT-TFs, cancer cells will respond to anticancer therapies differently, with greater sensitivity in epithelial cells.^125^

### TRAIL and resistance to anoikis in the metastatic context

The term ‘anoikis', from the Greek anoikos ‘without a home’, was proposed in the 1990s by Frisch and Francis^126^ to describe an apoptosis phenomenon following loss of cell-to-ECM interactions. The authors explained that anoikis occurs to abrogate an escape mechanism, meaning the possibility for a cell to reattach in an inappropriate tissue. This mechanism allows the limitation of oncogenic transformation without disrupting plasticity and cell migration necessary during development, repair and cell tissue homoeostasis. Anoikis and its resistance also increases the diversity of phenotypes.^127^ Thus, resistance to anoikis became a hallmark of malignant cells with their ability to grow under anchorage-independent conditions.^128^

In epithelial cells, anchorage to ECM represents an environmental signal that is mediated by integrins. Indeed, integrins β1 and β3 subunits, when in contact with ECM components, such as collagens, phosphorylate focal adhesion kinase (FAK), which, in turn phosphorylates Akt, leading to inhibition of pro-apoptotic proteins such as Bad. Consequently, the lack of ligation of integrins β1 and β3 subunits induces a decrease in both FAK protein and activity, but also those of the proto-oncogene tyrosine protein kinase Src or integrin-linked kinase (ILK), leading to the inhibition of the pro-survival Akt pathway.^128,129^

Evidence for a function of death receptors in anoikis has been described previously.^130^ When MDCK and HaCat cells lose their interactions with ECM, a caspase-8-dependent apoptotic cascade is triggered. This increasing caspase-8 activity after cell detachment occurs through FADD recruitment without DR4/DR5 activation, a process observed independently of the binding of death ligands.^131^ The authors also observed that Bcl-2 and Bcl-XL inhibit caspase-8-induced anoikis probably via a mitochondrial positive-feedback pathway by caspase-3. These data were further supported by another study showing that extrinsic apoptosis leading to anoikis was also triggered by caspase-8 in keratinocytes.^129^ This work revealed the positive feedback described above as a complementary interaction between the two apoptotic pathways. A negative post-transcriptional regulation of DR5 via miR126-3p was also proposed to explain the decrease in extrinsic apoptotic pathway signalling without affecting death receptor mRNA levels,^132^ but how TRAIL is associated with anoikis resistance during cancer progression remains unanswered.

Although DRs drive anoikis in normal cells, they fail to induce such a process in malignant cells, probably via a FLIP-dependent process.^133^ In breast cancer, cell anchorage suppresses TRAIL gene expression, whereas detachment increases its level. The autocrine role of endogenous TRAIL was then suspected to be associated with anoikis through activation of DR5 (and to a less extent DR4). Because the detached cells were found to be more sensitive to TRAIL, circulating tumour cells (CTCs) were considered as a potential target for TRAIL therapy.^134^ In fact, DR4/DR5 signalling allows caspase activation, leading to cleavage of Akt proteins and to their decreased expression levels. Because the Akt pathway plays a central role in mediating survival signalling, cell detachment via loss of integrin interactions with ECM is the first step in the inhibition of this anti-apoptotic signalling.^135^ In CRC cells, DR5 increases in cell suspension. The use of antagonists or DR5 knockdown is sufficient to inhibit anoikis, whereas no effects were observed concerning DR4. Exogenous TRAIL failed to increase anoikis as observed in a breast cancer model, and finally the proposed mechanism hypothesises that DR5 is activated by cross-linked soluble and membrane-bound TRAIL ligand.^128^

The mechanisms of anoikis resistance are numerous and they depend on the mutation status of the cancer cell model studied. Although one can suspect that a constitutive activation of pro-survival pathways could inhibit the apoptotic processes engaged after the loss of anchorage, thanks to acquired mutations, non-genetic heterogeneity associated with differences in protein expression levels can also largely impact the cell-fate decision. In the specific case of TRAIL-induced anoikis resistance, several mechanisms have been reported over the last two decades. For example, a decrease in DR4/DR5 expression has been described to explain such a resistance. In hepatoma cells, a low level of DR4/DR5 expression was associated with resistance of the TRAIL-induced apoptotic cascade even if upregulation of TRAIL mRNA was observed.^136^ Yet, no modulation in DR4/DR5 expression was observed between attached and detached human colon epithelial cells where TRAIL resistance was shown. Only increases in FAK and ILK activities and, secondly, the activation of the downstream Akt pathway, protect colon cells from TRAIL-induced apoptosis.^137^ Similar conclusions were reported in an ovarian model^138^ and in HL-60 cells.^139^ Interestingly, FAK not only stimulates the Akt pathway activation, but also interacts with caspase-8 in an adhesion-dependent manner, thus blocking the apoptotic extrinsic pathway in this condition;^140,141^ however, how TRAIL interacts with the integrin/FAK/Akt pathway remains unclear. More recently, TRAIL was described as a mediator of FAK signalling in the regulation of entosis (an invasion process involving two cells, where one is merging via the cytoplasm with the other) and necrosis in primary human mammary epithelial cells.^127^ Indeed, during detachment-induced cell death, even if TRAIL is rapidly increasing and this is for a long time (from 3 h to 72 h), FAK successfully inhibits TRAIL and protects cells during all of the processes.

Generally, the mechanisms of anoikis resistance linked to TRAIL treatment are shared with common apoptotic resistance mechanisms, especially those that interact with the extrinsic pathway. Indeed, a decrease in caspase-8 expression and its activity is associated with TRAIL resistance.^142^ Modulation of c-FLIP protein levels, the main endogenous pro-caspase-8 inhibitor^143^ and also an increase in the IAP protein family^144^ are other targets and regulators of this TRAIL-dependent resistance.

### TRAIL regulates the PD-L1-dependent immunogenic response

In lung cancer or melanoma, programmed cell death protein-1 (PD-1)/programmed death-ligand 1 (PD-L1) expression and activation is an indicator of poor prognosis for patients,^145,146^ but their inhibition has become a strategy to stimulate the immune response and increase cell death.^147,148^ There is a growing body of evidence suggesting intricate regulation processes between TRAIL and PD-L1 expression. In 2010, Tu et al. analysed the effect of the hepatitis C virus core protein (HCVc) on human liver and especially on innate immune Kupffer cells (KCs). They found that it was able to induce the upregulation of PD-L1 under interleukins (IL-1β–IL10) and TNF-α secretion, along with the inhibition of the cell-surface expression of the cytotoxic molecule TRAIL, a process dependent on the activation of the PI3K/Akt pathway.^149^ Moreover, in chronic lymphocytic leukaemia (CLL), the therapeutic agent trabectedin induces apoptosis of both human primary leukaemic cells, selected myeloid and lymphoid immunosuppressive cells mainly through the TRAIL/TNF pathway. In parallel, trabectedin also blocks the PD-1/PD-L1 axis by targeting PD-L1^+^ CLL cells, PD-L1^+^ monocytes/macrophages and PD-1^+^ T cells.^150^ Complementary data were reported in murine melanoma^151^ and in hepatocellular carcinoma cells.^152^ Even if this association is not completely understood, we now know that IFN-γ, a cytokine responsible for the increase in expression of PD-L1, can also sensitise cancer cells to TRAIL-mediated apoptosis through downregulation of c-FLIP.^153,154^ Based on the relationship between immune cells of the tumour microenvironment and cancer cells, a very attractive approach has been proposed using a bifunctional fusion protein, designated anti-PD-L1:TRAIL,^155^ that successfully targets both immune cells (myeloid effector cells and T-cell activity) and cancer cells sensitised by this method.^156^

EMT plays a central role in immunogenicity. It has been shown to promote metastasis via immunosuppression,^157,158^ but evidence that PD-L1 overexpression correlates with the induction of EMT has been demonstrated in non-small-cell lung carcinoma (NSCLC) and more recently in breast cancer via a ZEB-1/miR-200 mechanism.^159,160^ Upstream of this signalling cascade, glycogen synthase kinase (GSK)-3β/β-catenin controls the ZEB-1/miR-200 axis and allows β-catenin nuclear translocation under the negative control of SDH5, a succinate dehydrogenase component of the tricarboxylic acid cycle.^161,162^ In NSCLCs, EMT specifically regulates PD-L1 expression with the need of epigenetic reprogramming, thus leading to immune escape.^163^ This mechanism requires both demethylation of the PD-L1 promoter due to TGF-β action and activation of NF-κB via TNF-α stimulation, but is not accompanied by an increase in DR4/DR5 or TRAIL expression,^164^ suggesting that an inversely proportional relationship between the expression of PD-L1 and the increase in resistance to TRAIL dependent on the decrease in DR expression would occur under the control of EMT. This proposed mechanism has also been observed in glioblastoma (GBM) where cannabidiol (CBD) upregulated the gene and protein expression of DR5/TRAIL-R2 and sensitises GBM cells to TRAIL-induced apoptosis. The authors observed that, as expected, CBD caused a notable decrease in GBM surface levels of PD-L1.^165^

Different regulation pathways have been proposed to explain the simultaneous expression of TRAIL receptors and PD-L1. In tumour IFN-driven resistance, stimulation of cancer cells by IFN-γ leads to the nuclear translocation of signal transducer and activator of transcription 1 (STAT1). The activation of the IFN-γ/STAT1 axis is then responsible for the increase in PD-L1 and in parallel the decrease in TRAIL-R2.^166,167^ Blockade of the IFN-γ receptor in this same resistant model leads to the increase in TRAIL-R2 and allows natural killer (NK) cells to stimulate extrinsic apoptosis in the cancer cells. Another regulation highlighting the role of miRNA-429 in PD-L1 expression and TRAIL sensitivity has been recently described. Indeed, miR-429 is a member of the miR-200 family that can inhibit ZEB-1/2 or PTEN/Akt upregulation making this miRNA an EMT regulator.^168^ In gastric cancer, PD-L1 is positively correlated with TRAIL resistance where miR-429 is downregulated.^169^ The authors observed that miR-429 targets the 3′ untranslated region (UTR) of PD-L1. They proposed a mechanism where PD-L1 interacts with phosphorylated epidermal growth factor receptor (EGFR), leading to the activation of the pro-survival Akt pathway, thus blocking the TRAIL-dependent apoptotic process.

Finally, in KRAS-mutated cancer cells, oncogenic RAS allows the stabilisation of PD-L1 mRNA, leading to its increase and escape from immunosurveillance. This phenomenon partly accounts for the chemotherapeutic resistance observed. Interestingly in pancreatic ductal adenocarcinoma (PDCA), cancer cells also express endogenous TRAIL with an autocrine function. Via DR5 activation, TRAIL stimulates the migration and invasion of KRAS-mutated cancer cells in a Rac1-dependent manner. Knowing that Rac1 is usually inhibited via Rho-associated protein kinase (ROCK) under the control of KRAS in normal conditions, the authors proposed a new strategy to target both KRAS and TRAIL to stimulate the immunogenic response and increase patient survival.^170^

### Death receptor expression in circulating tumour cells (CTCs/CTMs)

CTCs are considered as putative precursors that might contribute, alone or in clusters, to cancer cell dissemination in the body, leading to metastasis.^171,172^ This cancer progression step is often called ‘the leukaemic phase’ of solid tumours as suggested by Mocellin et al.^173^ In patients’ blood, not only are CTCs collected but also apoptotic CTCs and CTC clusters described as circulating tumour microemboli (CTM) with higher metastatic potential. Together, they represent poor prognostic and pharmacodynamic biomarkers of solid tumours.^174**–**177^ Remarkably, only a small proportion of CTCs can give rise to metastasis.^178,179^

Anoikis resistance appears to be critical for the aetiology of CTCs.^180**–**182^ CTCs from prostate cancer cells lose their adhesive capacity through downregulation of E-cadherin, γ-catenin and β4 integrin with, in parallel, the gain of anti-apoptotic mechanisms increasing their resistance to cytotoxic stresses induced by immune cells.^183^ Among them, the authors observed a decrease in heat-shock protein 90β family member 1 (HSP90B1), a chaperone protein that not only enables escape from immune surveillance, but also increases Bcl-2 under the control of Akt pathway signalling activation. In another model, namely pancreatic cancer cells, Wnt2 was proposed as a candidate CTC gene. Wnt2 has been shown to be responsible for anoikis resistance through the activation of the non-canonical WNT/TAK1/IFN1 signalling pathway.^184^ Such examples are emerging in the literature, but all have a double signature in common: the decrease in epithelial markers and the gain of anti-apoptotic capacities as observed during EMT.

In CTCs from breast cancer, the molecular features of EMT were found inversely correlated with TRAIL plasma cytokine expression.^185^ Unfortunately, DR expression levels were not reported in this study. However, it seems that soluble TRAIL could have only weak apoptotic effects on CTCs independently of the DR concentrations as observed in a computational model.^186^ Different regulatory processes were proposed to understand DR modulations in CTCs, such as the c-Jun N-terminal kinase (JNK) pathway. In pancreatic CSCs, JNK inhibition allows the decrease in DcR1 via an IL-8-dependent autocrine process, while DR4/5 expression is increased, thereby sensitising cells to TRAIL treatment.^187^ Consequently, the authors observed diminished tumour burden and number of CTCs. Autophagic processes have also been shown to regulate sensitivity of CTCs to TRAIL,^188^ and to protect invasive cancer cells from anoikis.^189,190^ In a breast cancer cell line, DR4/5 expression is decreased in cell suspension in contrast to adherent cells, thus increasing TRAIL resistance. Mechanistically, DR4/5 undergo a rapid endocytosis, sequestration in the nucleus and degradation in the autophagosome.^191^

Given that EMT provides mesenchymal cells with the ability to resist to apoptosis, anoikis and some stem cell characteristics (regulated by different factors such as TGF-β, Wnt or Notch^192^), more evidence is needed to evaluate whether death receptor agonists could favour the emergence of CTCs through EMT mechanisms and further assess the sensitivity of CTCs to these drugs.

## Conclusions and perspectives

Activation of death receptors allows pleiotropic effects whether related to cell death (apoptosis, necrosis, necroptosis, pyroptosis…) or to survival (differentiation, division, migration, entosis, EMT…). However, cell fate will ultimately depend on a wide range of environmental and cell contexts with both genetic and non-genetic variations. This response heterogeneity is at the origin of cell resistance, an adaptive mechanism that impairs cancer drug development and therapeutic strategies.^193^ In this review, we examined how EMT participates to increase this response heterogeneity which, in turn, enhances cancer cell survival. There are other possible mechanisms by which EMT could increase response heterogeneity through interactions with the tumour microenvironment. First, cancer cell growth is usually accompanied with a decrease in the availability of oxygen and other necessary elements within the tumour. This transient ischaemia stimulates the expression of the hypoxia-inducible factor family (HIF-1) that mediates the angiogenic response and controls different EMT-TFs (TCF3, ZEB-1/2 and TWIST1) responsible for E-cadherin downregulation.^194,195^ Secondly, carcinoma-associated fibroblasts (CAFs) are stroma cells that secrete soluble TGF-β, matrix metalloproteinases (MMPs), hepatocyte growth factor (HGF) and urokinase-type plasminogen activator (uPA). These CAFs are also recruited and activated from resident fibroblasts via the equivalent secretion of factors produced by cancer cells in EMT.^196^ Finally, inflammation stimulates and maintains EMT through production of cytokines (TGF-β, TNF-α, IL-1β, IL-6, IL-8, chemokine (C–X–C motif) ligand 1 (CXCL1) and CC chemokine ligand 18 (CCL18)) by infiltrating immune cells, including tumour-associated macrophages (TAMs) and lymphocytes.^197,198^ Because the EMT programme is regulated temporally and spatially (activation at the invasive front of the tumour), the differential communication between cancer cells and the microenvironment can further contribute to increase response heterogeneity to drug treatments.^29,199^

## Data Availability

Not applicable.
